# On the Composition of Air at Different Heights in Close Apartments

**Published:** 1846-12

**Authors:** 


*On the Composition of Air at different heights in close Apartments.*—Lassaigne has drawn the following conclusions from a series of experiments on this subject:

1. In rooms where the air is confined, and has been respired for some time without renewal, the carbonic acid expired is not found exclusively, as some have asserted, in the lower strata.

2. In accordance with the laws of physics, confirmed by experiment, the carbonic acid is nearly equally diffused throughout the whole volume of confined air which has been respired by a certain number of persons.

3. The slight differences observed would lead to the inference, that the proportion of carbonic acid was, under these circumstances, greater in the upper strata, were it not that the differences may depend upon errors in the quantitative estimation of the gaseous components of the air.

4. These facts show the erroneous principles upon which some modern theories of ventilation are based: for it is clear that the *whole* mass of air which has been respired by many persons requires renewal, so that the vitiated air may be entirely expelled.

5. The uneasiness experienced from respiring the heated air in the upper part of crowded and badly ventilated theatres, is due rather to its rarefaction than to its chemical composition; for the latter is almost identical in the upper and lower strata. The acts of respiration are more (?) full and frequent when rarified air is breathed; hence certain physiological effects are induced which are not observed in the respiration of air at the common temperature.—*Lon. Med. Gaz.*, from *Comptes Rendus.*—*Am. Jour. Med. Sci.*

